# Risk factors for early neurological deterioration in patients with acute ischaemic stroke and assessment of short-term prognosis

**DOI:** 10.3389/fneur.2026.1845703

**Published:** 2026-05-22

**Authors:** Ming Peng, Yanqing Zhang

**Affiliations:** Department of Neurology, Sinopharm Tongmei General Hospital, Datong, Shanxi, China

**Keywords:** acute ischaemic stroke, early neurological deterioration, NIHSS score, prognostic assessment, risk factors

## Abstract

**Objective:**

To identify independent risk factors for early neurological deterioration (END) in patients with acute ischaemic stroke (AIS) and evaluate its impact on short-term clinical outcomes.

**Methods:**

A total of 186 AIS patients admitted between January 2023 and January 2025 were retrospectively enrolled and divided into an END group (*n* = 62) and a non-END group (*n* = 124), based on a NIHSS score increase of ≥2 points within 72 h. Baseline characteristics, laboratory parameters, neuroimaging features, and treatment details were compared. Multivariable logistic regression identified independent risk factors for END and for unfavorable outcomes among END patients. Neurological function was assessed by serial NIHSS scores, and 3-month prognosis by the modified Rankin Scale (mRS).

**Results:**

The END group showed significantly higher admission NIHSS score, fasting plasma glucose, glycated hemoglobin, homocysteine, high-sensitivity C-reactive protein (hs-CRP), D-dimer, and greater proportions of hypertension, diabetes mellitus, atrial fibrillation, large artery atherosclerosis, cardioembolic subtypes, and large infarction (all *P* < 0.05). Multivariable analysis identified higher admission NIHSS score, fasting plasma glucose, hs-CRP, D-dimer, atrial fibrillation history, and large infarction as independent risk factors for END. END patients had persistently elevated NIHSS scores, higher rates of unfavorable 3-month outcomes (mRS ≥3), longer hospital stays, and greater mortality (all *P* < 0.05). Among END patients, admission NIHSS ≥12, large infarction, and hs-CRP ≥15 mg/L independently predicted unfavorable outcomes.

**Conclusions:**

Higher admission NIHSS score, fasting plasma glucose, hs-CRP, D-dimer, atrial fibrillation, and large infarction are independent risk factors for END in AIS. END is associated with worse neurological recovery, prolonged hospitalization, and higher 3-month mortality. Early identification of high-risk patients and targeted intervention are essential for improving outcomes.

## 1 Introduction

Acute ischaemic stroke (AIS) is the most prevalent subtype of stroke, accounting for the majority of all stroke cases (1, 2). It is characterized by high incidence, high rates of disability, and high mortality, and represents a leading cause of disease burden among the Chinese population. Early neurological deterioration (END) refers to the progressive worsening of neurological deficits occurring in the early phase of AIS, typically defined as an increase in NIHSS score of ≥2 points within 72 h of admission (3). Published literature suggests that END occurs in approximately 20%−40% of patients with AIS and constitutes an important determinant of clinical outcome (4).

The pathophysiological mechanisms underlying END are complex and may involve expansion of the ischaemic penumbra, thrombus propagation, cerebral oedema, haemorrhagic transformation, and systemic factors such as infection and metabolic disturbances (5). Prior studies have consistently demonstrated that END is associated with significantly higher rates of disability and mortality, prolonged hospitalization, and increased healthcare costs compared with patients without END, thereby imposing a substantial burden on patients and healthcare systems (6). Early identification of risk factors predisposing patients to END and prompt initiation of targeted therapeutic strategies are therefore of considerable clinical importance for improving outcomes in AIS.

Although a number of studies have investigated risk factors for END in AIS, reported findings have been inconsistent across studies. Several investigations have been limited by small sample sizes and a narrow range of included variables, and few studies have combined risk factor analysis with comprehensive prognostic evaluation in a single dataset. To address these gaps, this study retrospectively analyzed clinical data from 186 patients with AIS treated at our institution, aiming to systematically delineate risk factors for END, evaluate the effect of END on short-term prognosis, and identify determinants of unfavorable outcomes among patients with END, thereby providing a basis for early clinical recognition and intervention.

## 2 Methods

### 2.1 Study design

This was a retrospective case–control study approved by the Ethics Committee of Sinopharm Tongmei General Hospital (Approval No. 2023-K-28) and conducted in accordance with the Declaration of Helsinki. Patients with AIS admitted to the Department of Neurology of our institution between January 2023 and January 2025 were identified through the hospital information system.

Inclusion criteria were: (1) diagnosis of AIS confirmed by cranial computed tomography (CT) and/or magnetic resonance imaging (MRI) in accordance with the Chinese Guidelines for Diagnosis and Treatment of Acute Ischaemic Stroke (7); (2) onset-to-admission time ≤ 24 h; (3) age ≥18 years; (4) admission NIHSS score ≥1; and (5) complete clinical records with ≥2 NIHSS assessments within 72 h.

Exclusion criteria were: (1) haemorrhagic stroke or transient ischaemic attack; (2) severe cardiac, hepatic, or renal failure; (3) comorbid malignancy; (4) comatose state at admission (GCS ≤ 8); and (5) incomplete clinical data.

After applying these criteria, 186 patients were included. Patients were allocated to the END group (*n* = 62) if their NIHSS score increased by ≥2 points relative to the admission value within 72 h (8), and to the non-END group (*n* = 124) otherwise. The overall incidence of END was 33.33%. The ≥2-point threshold was selected based on evidence from multicenter studies confirming its clinical reproducibility and prognostic relevance (8), and in alignment with multiple domestic multicenter investigations to facilitate cross-study comparisons. Although a cutoff of ≥4 points has been used in some studies to improve specificity for purely ischemic deterioration, the ≥2-point criterion provides greater sensitivity for detecting early neurological changes, enabling timelier identification of high-risk patients, thus balancing diagnostic sensitivity with clinical applicability.

### 2.2 Data collection

The following data were extracted from the electronic medical record system: (1) general demographic information: age, sex, and body mass index (BMI); (2) medical history: hypertension, diabetes mellitus, coronary heart disease, atrial fibrillation, smoking, and alcohol use; (3) vital signs at admission: systolic and diastolic blood pressure; (4) admission laboratory investigations: fasting plasma glucose, glycated hemoglobin (HbA1c), total cholesterol, triglycerides, low-density lipoprotein cholesterol (LDL-C), high-density lipoprotein cholesterol (HDL-C), homocysteine, hs-CRP, D-dimer, fibrinogen, white blood cell count, and platelet count; (5) neuroimaging data: TOAST classification, infarct location, and infarct size (large infarct was defined as an infarct involving more than one-third of the ipsilateral middle cerebral artery territory on CT or MRI, or an Alberta Stroke Program Early CT Score (ASPECTS) of ≤ 5); and (6) treatment details: intravenous thrombolysis, antiplatelet therapy, and statin use.

### 2.3 Outcome measures

#### 2.3.1 Primary outcomes

(1) Risk factors for END: independent risk factors were identified by comparing clinical characteristics between the END and non-END groups using univariable and multivariable logistic regression. (2) Neurological function: the NIHSS was used to assess the severity of neurological deficits at admission, 72 h, day 7, and discharge. (3) Short-term prognostic assessment: functional outcome at 3 months was evaluated using the modified Rankin Scale (mRS), with mRS scores 0–2 defined as favorable outcome and mRS scores 3–6 defined as unfavorable outcome (9).

#### 2.3.2 Secondary outcomes

Secondary outcomes included (1) length of hospital stay, (2) discharge status, and (3) risk factors for unfavorable prognosis in END patients.

#### 2.3.4 Statistical analysis

Statistical analyses were performed using SPSS version 26.0. Continuous variables were tested for normality with the Shapiro–Wilk test; normally distributed data are presented as mean ± standard deviation and were compared using the independent-samples *t*-test. Categorical variables are expressed as frequency and percentage [*n* (%)] and were compared using the chi-squared test or Fisher's exact test, as appropriate. Univariable and multivariable logistic regression analyses were employed to identify risk factors for END. A two-sided *P*-value < 0.05 was considered statistically significant.

## 3 Results

### 3.1 Baseline characteristics

Compared with the non-END group, the END group had significantly higher mean age, greater proportions of hypertension, diabetes mellitus, and atrial fibrillation, higher admission NIHSS score, and lower admission GCS score (all *P* < 0.05). No significant differences were observed between groups in sex, BMI, history of coronary heart disease, smoking, alcohol use, systolic blood pressure, or diastolic blood pressure (all *P* > 0.05). Full results are presented in Table 1.

**Table 1:** Comparison of baseline characteristics between groups (mean ± SD/[*n* (%)]).

Variable	END group (*n* = 62)	Non-END group (*n* = 124)	t/χ^2^	*P*-value
Mean age (years)	69.47 ± 10.92	66.02 ± 10.66	2.064	0.040
Male/female (*n*)	31/31	68/56	0.389	0.533
BMI (kg/m^2^)	23.94 ± 3.87	23.87 ± 3.51	0.118	0.906
Hypertension history [*n* (%)]	48 (77.42)	70 (56.45)	7.835	0.005
Diabetes mellitus history [*n* (%)]	32 (51.62)	40 (32.26)	6.526	0.011
Coronary heart disease history [*n* (%)]	14 (22.58)	25 (20.16)	0.146	0.702
Atrial fibrillation history [*n* (%)]	23 (37.10)	21 (16.94)	9.303	0.002
Smoking history [*n* (%)]	26 (41.94)	50 (40.32)	0.044	0.833
Alcohol use history [*n* (%)]	18 (29.03)	36 (29.03)	0.000	1.000
Onset-to-admission time (h)	10.67 ± 7.06	11.18 ± 6.71	0.787	0.376
Admission NIHSS score	10.69 ± 4.23	6.73 ± 3.69	6.562	<0.001
Admission GCS score	12.55 ± 1.48	13.90 ± 1.09	6.395	<0.001
Systolic blood pressure (mmHg)	149.98 ± 15.00	150.03 ± 19.99	0.018	0.987
Diastolic blood pressure (mmHg)	86.02 ± 9.99	85.84 ± 10.13	0.113	0.910

END, early neurological deterioration; NIHSS, National Institutes of Health Stroke Scale; GCS, Glasgow Coma Scale.

### 3.2 Admission laboratory parameters

The END group showed significantly higher fasting plasma glucose, HbA1c, homocysteine, hs-CRP, D-dimer, fibrinogen, and white blood cell count, and significantly lower HDL-C compared with the non-END group (all *P* < 0.05). No significant differences were found in total cholesterol, triglycerides, LDL-C, or platelet count (all *P* > 0.05). Results are shown in Table 2.

**Table 2:** Comparison of admission laboratory parameters between groups (mean ± SD).

Laboratory parameter	END group (*n* = 62)	Non-END group (*n* = 124)	*t*	*P*-value
Fasting plasma glucose (mmol/L)	8.29 ± 2.25	6.42 ± 1.69	5.770	<0.001
Glycated hemoglobin (%)	7.45 ± 1.33	6.32 ± 1.13	6.067	<0.001
Total cholesterol (mmol/L)	4.80 ± 1.05	4.76 ± 0.87	0.275	0.783
Triglycerides (mmol/L)	1.66 ± 0.69	1.68 ± 0.70	0.112	0.911
LDL-C (mmol/L)	2.82 ± 0.79	2.83 ± 0.75	0.089	0.929
HDL-C (mmol/L)	1.09 ± 0.27	1.20 ± 0.30	2.299	0.023
Homocysteine (μmol/L)	18.22 ± 4.92	14.08 ± 4.46	5.763	<0.001
hs-CRP (mg/L)	12.87 ± 11.48	4.42 ± 3.67	5.658	<0.001
D-dimer (mg/L)	1.82 ± 1.60	0.80 ± 0.62	6.244	<0.001
Fibrinogen (g/L)	3.70 ± 0.79	3.26 ± 0.65	4.042	<0.001
White blood cell count (×10^9^/L)	8.50 ± 2.51	7.13 ± 1.91	3.788	<0.001
Platelet count (×10^9^/L)	217.55 ± 62.63	225.05 ± 53.22	0.853	0.395

LDL-C = low-density lipoprotein cholesterol; HDL-C = high-density lipoprotein cholesterol.

### 3.3 Neuroimaging and treatment characteristics

The END group had a significantly higher proportion of large artery atherosclerosis and cardioembolic subtypes and a higher proportion of large infarcts compared with the non-END group (all *P* < 0.05). There were no significant differences between groups in infarct location, use of intravenous thrombolysis, antiplatelet therapy, or statins (all *P* > 0.05). Results are presented in Table 3.

**Table 3:** Comparison of neuroimaging and treatment characteristics between groups [*n* (%)].

Characteristic	END group (*n* = 62)	Non-END group (*n* = 124)	χ^2^	*P*-value
TOAST classification				
Large artery atherosclerosis	28 (45.16)	35 (28.23)	9.07	0.028
Small artery occlusion	13 (20.97)	50 (40.32)		
Cardioembolism	15 (24.19)	23 (18.55)		
Other/undetermined etiology	6 (9.68)	16 (12.90)		
Infarct location				
Anterior circulation	35 (56.45)	68 (54.84)	0.100	0.951
Posterior circulation	20 (32.26)	39 (31.45)		
Combined anterior and posterior	7 (11.29)	17 (13.71)		
Infarct size				
Large	25 (40.32)	22 (17.74)	11.890	0.003
Medium	17 (27.42)	43 (34.68)		
Small	20 (32.26)	59 (47.58)		
Intravenous thrombolysis [*n* (%)]	15 (24.19)	42 (33.87)	1.821	0.177
Antiplatelet therapy [*n* (%)]	54 (87.10)	107 (86.29)	0.023	0.879
Statin therapy [*n* (%)]	51 (82.26)	99 (79.84)	0.155	0.694

TOAST, trial of org 10172 in acute stroke treatment.

### 3.4 Univariable analysis of risk factors for END

Univariable analysis of clinical variables potentially associated with END identified the following factors as statistically significant: age, hypertension, diabetes mellitus, atrial fibrillation, admission NIHSS score, admission GCS score, fasting plasma glucose, HbA1c, HDL-C, homocysteine, hs-CRP, D-dimer, fibrinogen, white blood cell count, TOAST subtype, and large infarction (all *P* < 0.05). Results are shown in Table 4, which summarizes the above univariable findings to serve as a reference for variable selection in the subsequent multivariable analysis.

**Table 4:** Univariable analysis of risk factors for END in patients with AIS.

Variable	END group (*n* = 62)	Non-END group (*n* = 124)	t/χ^2^	*P*-value
Mean age (years)	69.47 ± 10.92	66.02 ± 10.66	2.064	0.040
Hypertension history [*n* (%)]	48 (77.42)	70 (56.45)	7.835	0.005
Diabetes mellitus history [*n* (%)]	32 (51.62)	40 (32.26)	6.526	0.011
Atrial fibrillation history [*n* (%)]	23 (37.10)	21 (16.94)	9.303	0.002
Admission NIHSS score	10.69 ± 4.23	6.73 ± 3.69	6.562	<0.001
Admission GCS score	12.55 ± 1.48	13.90 ± 1.09	6.395	<0.001
Fasting plasma glucose (mmol/L)	8.29 ± 2.25	6.42 ± 1.69	5.770	<0.001
Glycated hemoglobin (%)	7.45 ± 1.33	6.32 ± 1.13	6.067	<0.001
HDL-C (mmol/L)	1.09 ± 0.27	1.20 ± 0.30	2.299	0.023
Homocysteine (μmol/L)	18.22 ± 4.92	14.08 ± 4.46	5.763	<0.001
hs-CRP (mg/L)	12.87 ± 11.48	4.42 ± 3.67	5.658	<0.001
D-dimer (mg/L)	1.82 ± 1.60	0.80 ± 0.62	6.244	<0.001
Fibrinogen (g/L)	3.70 ± 0.79	3.26 ± 0.65	4.042	<0.001
White blood cell count (×10^9^/L)	8.50 ± 2.51	7.13 ± 1.91	3.788	<0.001
TOAST classification				
Large artery atherosclerosis	28 (45.16)	35 (28.23)	9.07	0.028
Small artery occlusion	13 (20.97)	50 (40.32)		
Cardioembolism	15 (24.19)	23 (18.55)		
Other/undetermined etiology	6 (9.68)	16 (12.90)		
Infarct size (large) [*n* (%)]	25 (40.32)	22 (17.74)	11.890	0.003

### 3.5 Multivariable logistic regression analysis of risk factors for END

Variables that reached significance in the univariable analysis (*P* < 0.05) were entered into the multivariable logistic regression model. Prior to model construction, multicollinearity was assessed using the variance inflation factor (VIF); a VIF > 10 was considered indicative of severe collinearity. Given the high collinearity observed between admission NIHSS score and GCS score, only the NIHSS score was retained in the multivariable model. The analysis identified higher admission NIHSS score, higher fasting plasma glucose, higher hs-CRP, higher D-dimer, history of atrial fibrillation, and large infarction as independent risk factors for END (all *P* < 0.05). Results are presented in Table 5.

**Table 5:** Multivariable logistic regression analysis of risk factors for END in patients with AIS.

Variable	B	SE	Wald χ^2^	*OR*	95% CI	*P*-value
Admission NIHSS score	0.218	0.048	20.626	1.244	1.132–1.367	<0.001
Fasting plasma glucose	0.296	0.092	10.348	1.344	1.122–1.610	0.001
hs-CRP	0.078	0.028	7.762	1.081	1.023–1.142	0.005
D-dimer	0.612	0.196	9.754	1.844	1.256–2.708	0.002
Atrial fibrillation history	0.826	0.372	4.926	2.284	1.102–4.736	0.026
Large infarction	0.918	0.386	5.656	2.504	1.175–5.338	0.017

B, regression coefficient; SE, standard error; OR, odds ratio; CI, confidence interval.

### 3.6 NIHSS scores at different time points

Repeated-measures analysis of variance demonstrated significant time effects, group effects, and time-by-group interaction effects (all *P* < 0.05). NIHSS scores at all assessed time points were significantly higher in the END group than in the non-END group (all *P* < 0.05). In the END group, NIHSS scores peaked at 72 h and subsequently declined but remained elevated at discharge. Results are shown in Table 6.

**Table 6:** NIHSS scores at different time points (mean ± SD, points).

Time point	END group (*n* = 62)	Non-END group (*n* = 124)	*t*	*P*-value
Admission	10.69 ± 4.23	6.73 ± 3.69	6.562	<0.001
72 h	14.95 ± 4.63	6.82 ± 3.77	12.826	<0.001
Day 7	11.98 ± 4.71	4.46 ± 3.51	12.245	<0.001
Discharge	9.90 ± 5.12	2.87 ± 2.95	11.879	<0.001

NIHSS, National Institutes of Health Stroke Scale; END, early neurological deterioration.

### 3.7 Three-month outcomes

The proportion of patients with non-fatal unfavorable outcomes (mRS 3–5) and the total unfavorable outcome rate (mRS ≥3) at 3 months were both significantly higher in the END group than in the non-END group (*P* < 0.05). The END group also had significantly longer hospital stays (*P* < 0.05). Mortality at 3 months (mRS = 6) was 16.13% in the END group compared with 4.84% in the non-END group (*P* < 0.05). Detailed results are presented in Table 7.

**Table 7:** Comparison of 3-month outcomes between groups (mean ± SD / [*n* (%)]).

Outcome measure	END group (*n* = 62)	Non-END group (*n* = 124)	t/χ^2^	*P*-value
Length of hospital stay (days)	17.44 ± 5.79	11.85 ± 3.68	6.919	<0.001
mRS score	3.87 ± 1.49	2.20 ± 1.61	6.826	<0.001
Favorable outcome (mRS 0–2) [*n* (%)]	10 (16.13)	77 (62.10)	35.082	<0.001
Non-fatal unfavorable outcome (mRS 3–5) [*n* (%)]	42 (67.74)	41 (33.06)	20.114	<0.001
Death (mRS 6) [*n* (%)]	10 (16.13)	6 (4.84)	6.701	0.010
Total unfavorable outcome (mRS ≥3) [*n* (%)]	52 (83.87)	47 (37.90)	—	—
Discharge status				
Improved	10 (16.13)	77 (62.10)	37.890	< 0.001
Unchanged	31 (50.00)	35 (28.23)		
Deteriorated	11 (17.74)	6 (4.84)		
Death	10 (16.13)	6 (4.84)		

mRS, modified Rankin Scale; END, early neurological deterioration.

‘—' denotes that no formal test was applied as this row is the complement of the favorable outcome row.

### 3.8 Multivariable logistic regression analysis of risk factors for unfavorable outcomes in END patients

Using the 62 END patients as the study population, with unfavorable 3-month outcome (mRS ≥3) as the dependent variable, candidate variables with potential prognostic relevance were first screened by univariable analysis. Given the limited sample size of the favorable-outcome subgroup (*n* = 10), only the three variables with the most significant differences in the univariable analysis were included in the multivariable model to avoid overfitting. Admission NIHSS score ≥12, large infarction, and hs-CRP ≥15 mg/L were identified as independent risk factors for unfavorable outcomes in END patients (all *P* < 0.05). Results are shown in Table 8.

**Table 8:** Multivariable logistic regression analysis of risk factors for unfavorable outcomes in END patients.

Variable	B	SE	Wald χ^2^	*OR*	95% CI	*P*-value
Admission NIHSS score ≥12	1.286	0.482	7.122	3.618	1.407–9.306	0.008
Large infarction	1.142	0.468	5.956	3.133	1.252–7.842	0.015
hs-CRP ≥15 mg/L	1.068	0.452	5.586	2.910	1.200–7.058	0.018

B, egression coefficient; SE, standard error; OR, odds ratio; CI, confidence interval; hs-CRP, high-sensitivity C-reactive protein.

## 4 Discussion

### 4.1 Incidence and risk factors for END in AIS

In the present study, END occurred in 33.33% (62/186) of patients with AIS, which is broadly consistent with the reported range of 20%−40% in the existing literature (10). Multivariable logistic regression identified higher admission NIHSS score, higher fasting plasma glucose, elevated hs-CRP, elevated D-dimer, history of atrial fibrillation, and large infarction as independent risk factors for END.

Among these, the admission NIHSS score was among the strongest predictors of END; in this cohort, each one-point increase in NIHSS score was associated with a 24.4% higher odds of END (*OR* = 1.244). Prior studies have similarly reported that a higher admission NIHSS score reflects more severe neurological deficits and more extensive ischaemic injury, with a potentially larger ischaemic penumbra that is more susceptible to progression to irreversible damage within a short timeframe, thereby predisposing to END (11, 12). These findings suggest that patients presenting with higher NIHSS scores warrant more frequent neurological monitoring to facilitate early detection of END.

Hyperglycaemia was also an important risk factor for END in this cohort, with each 1 mmol/L increase in fasting plasma glucose associated with a 34.4% higher odds of END (*OR* = 1.344). We propose that hyperglycaemia may exacerbate ischaemic brain injury through several mechanisms: first, by promoting anaerobic glycolysis, leading to intracellular lactate accumulation and acidosis that augments neuronal injury (13); second, by amplifying oxidative stress and inflammatory responses, thereby compromising blood–brain barrier integrity (14); and third, by impairing the collateral compensatory capacity within the ischaemic penumbra (15). These observations suggest that rigorous glycaemic control may be an important strategy for reducing the risk of END.

The present study identified evidence implicating systemic inflammation in the development of END. Elevated hs-CRP was an independent risk factor for END (*OR* = 1.081), indicating that systemic inflammatory responses contribute to the pathophysiology of END. During the acute phase of AIS, ischaemic tissue releases large quantities of inflammatory mediators that activate microglia and peripheral immune cells, triggering inflammatory cascades that further damage the ischaemic penumbra and promote infarct expansion (16).

It is further worth emphasizing that several of the risk factors identified above, namely hyperglycaemia, elevated admission NIHSS score, and atrial fibrillation, are all amenable to targeted intervention in Chinese clinical practice. First, glycaemic management can be implemented through optimisation of post-admission hypoglycaemic regimens combined with bedside glucose monitoring. Second, in patients with higher NIHSS scores, increased frequency of early neurological assessment is recommended to facilitate timely detection of END. Third, in patients with comorbid atrial fibrillation, prompt evaluation of anticoagulation indications should be initiated to mitigate the risk of END attributable to cardioembolism. These findings carry practical implications for stroke management in primary and secondary care hospitals across China.

### 4.2 D-dimer and END

D-dimer, a fibrin degradation product, is a sensitive marker of coagulation system activation and fibrinolytic activity (17). In the present study, elevated D-dimer was an independent risk factor for END (*OR* = 1.844), indicating that coagulopathy and thrombus propagation play important roles in the pathogenesis of END. Elevated D-dimer levels may reflect ongoing thrombus formation and lysis, suggesting that intravascular thrombi remain unstable or that new thrombus formation is occurring. Combined with the presence of atrial fibrillation, another independent risk factor identified in this study (*OR* = 2.284), this suggests that cardioembolism may contribute to END through recurrent microemboli or fragmentation of emboli, leading to progressive expansion of the ischaemic territory (18). Clinically, patients with AIS and elevated D-dimer should be monitored closely for evidence of thrombus propagation, and antithrombotic regimens should be adjusted as indicated. It should be noted that both hs-CRP and D-dimer undergo dynamic changes during the acute phase of AIS; however, the present study relied solely on single baseline measurements obtained at admission without serial monitoring, which may limit the ability to fully capture the relationship between temporal trends in these biomarkers and disease progression. The clinical predictive value of serial measurements at defined time points, such as admission, 24 h, and 72 h, warrants further investigation in future studies. In clinical practice, repeat testing within 72 h is recommended for patients presenting with markedly elevated hs-CRP or D-dimer at admission, to assist in the assessment of disease trajectory.

### 4.3 Impact of END on prognosis

The present study demonstrated that patients who developed END had substantially worse short-term outcomes compared with those without END. Specifically, the 3-month unfavorable outcome rate and mortality were both significantly higher in the END group, and END patients had longer hospital stays, highlighting additional concerns regarding healthcare resource utilization and the risk of nosocomial infection. The longitudinal NIHSS data confirm the severe impact of END on neurological function: scores in the END group peaked at 72 h and, although they subsequently declined, remained markedly elevated at discharge, suggesting that the neurological deficits caused by END are unlikely to recover fully within the short term. In contrast, NIHSS scores in the non-END group showed a sustained declining trend, with a pronounced reduction by discharge, indicating a more favorable trajectory of neurological recovery. It is worth noting that NIHSS scores in the non-END group showed a marginal increase at 72 h compared with admission, which did not approach the END threshold; this may be attributable to transient worsening of cerebral oedema in the early disease course or to inter-rater variability.

### 4.4 Risk factors for unfavorable outcomes in END patients

Further analysis of determinants of unfavorable outcomes among END patients identified admission NIHSS score ≥12, large infarction, and hs-CRP ≥15 mg/L as independent risk factors. Patients with severe neurological deficits at admission likely sustain more extensive cerebral tissue injury, reducing compensatory capacity; when END is superimposed, further neurological deterioration may occur, leading to worse outcomes (19). Large infarction implies greater loss of functional brain tissue, limited availability of collateral circulation, and reduced potential for neurological recovery (20). Persistently elevated hs-CRP suggests sustained inflammatory activity after admission, which may augment secondary brain injury and impair the reparative process (21). These findings suggest that, among patients who have already developed END, admission NIHSS score, infarct size, and inflammatory markers may serve as valuable prognostic indicators to guide early risk stratification and the adoption of more aggressive therapeutic and rehabilitation strategies.

### 4.5 Limitations

Several limitations of the present study should be acknowledged. First, this was a single-center retrospective study with a relatively small sample size, and the generalisability of findings requires validation in multicentre prospective studies. Second, END was defined as an increase in NIHSS score of ≥2 points; use of alternative definitional thresholds may yield different results. Third, mechanical thrombectomy and endovascular treatment were not routinely performed at our center during the study period; the absence of these data may introduce confounding in the analysis of END among patients with large vessel occlusion subtypes, and future studies should include patients treated with endovascular intervention to evaluate its influence on END. Fourth, the specific aetiological subtypes underlying END in the 62 affected patients, such as haemorrhagic transformation, cerebral oedema, and infarct expansion, were not systematically classified, which may limit in-depth analysis of the pathological mechanisms driving END. Fifth, both hs-CRP and D-dimer were measured only once at admission without serial monitoring, which may not fully capture their dynamic changes during the acute phase. Finally, the multivariable analysis of prognostic factors among END patients was constrained by a small favorable-outcome subgroup (*n* = 10), resulting in a low events-per-variable ratio; the stability of the model findings requires confirmation in studies with larger sample sizes. Future prospective multicentre studies with larger sample sizes are warranted to further validate the conclusions of the present study.

## 5 Conclusions

Higher admission NIHSS score, higher fasting plasma glucose, elevated hs-CRP, elevated D-dimer, history of atrial fibrillation, and large infarction are independent risk factors for END in patients with AIS. Patients who develop END have worse neurological outcomes, longer hospital stays, and substantially higher rates of unfavorable prognosis and mortality at 3 months compared with those without END. Among patients with END, admission NIHSS score ≥12, large infarction, and hs-CRP ≥15 mg/L are independent risk factors for unfavorable outcomes. Clinicians should prioritize early dynamic neurological assessment in patients with AIS, identify high-risk individuals in a timely manner, and implement targeted interventions including rigorous glycaemic control, reduction of systemic inflammation, and appropriate antithrombotic therapy to reduce the incidence of END and improve patient outcomes.

## Data Availability

The original contributions presented in the study are included in the article/supplementary material, further inquiries can be directed to the corresponding author.
